# Reflection on the past and looking into the future—a celebration of 10 years of *BJR*|*Case Reports*

**DOI:** 10.1093/bjrcr/uaae010

**Published:** 2024-03-22

**Authors:** Giulia A Zamboni

**Affiliations:** Institute of Radiology, Department of Diagnostics and Public Health, University of Verona, Verona 37134, Italy

The 10th volume of *BJR*|*Case Reports*, along with the transition to our new publishing partner, Oxford University Press, in January 2024 is the occasion for looking back and celebrating the history of our journal, and to envision its future.

Case reports have a long history, as long as the history of medicine. In radiology, as in many other disciplines, they were for decades one of the most important forms of publication, with colleagues sharing cases and contributing to the exponential growth of our discipline. Over time this has of course changed, together with the universally recognized necessity of using robust scientific methods and evidence-based medicine. Still, recent years, and in particular during the COVID-19 pandemic, we have dramatically been reminded of the importance of sharing new cases in a timely fashion, faster than what is allowed by acquiring the data and analysing it to produce a research article.

Many journals, including *BJR*, have ceased publication of case reports. There was and still is, however, definite interest from the radiological and oncological community in sharing interesting and unique cases, from which all can learn, both the seasoned consultant and those in training. For colleagues in training, case reports are often the first approach to writing a scientific paper, and it is important that there are high quality destinations for their efforts.

To cover this need, *BJR*|*Case Reports* was launched in 2015 by the British Institute of Radiology (BIR), and the first inaugural issue was published in March 2015. The founding Editor-in-Chief was Professor Giuseppe Guglielmi, who contributed to creating a multidisciplinary, international case reports journal which he led for 6 years. With a truly international Editorial Board, strong ethics and a rigorous peer-review system, *BJR*|*Case Reports* is now one of the most reputed journals dedicated to case reports on medical imaging and radiation oncology. The choice of creating an open access publication was due to the growing demand for open access publishing outlets.

After its creation, thanks to Professor Guglielmi’s guidance and the support of the BIR office staff, the publisher and the BIR, the journal grew and in 2017 it was indexed in the Directory of Open Access Journals and then awarded the seal of best practice in open access. This led to its being indexed in PubMed in 2018, a fundamental step towards the growth and the diffusion of the journal. Today *BJR*|*Case reports* is accessed all over the world, and publishes articles written by authors based in 80 countries.

After being Senior Editor for the gastrointestinal imaging section of the journal, I was fortunate to be chosen as Professor Guglielmi’s successor and became the new Editor-in-Chief in January 2021. I have therefore been able to witness the growth and maturity of the journal, which over time has increased its success and readership.

The number of submissions skyrocketed in 2020, with a 62% increase on 2019 due to the pandemic and increased even more in 2021. Even with a small decline, the number of submissions in 2023 was still above the pre-pandemic volume, showing the continuous interest of the radiological community in case reports. In 2021 the journal increased its number of issues from four to six issues per year, to accommodate the large number of submissions. This increase in published issues was accomplished without compromising the high quality of the content that we provide to our readers, thanks to the quality work provided by the Editorial Board, the BIR editorial office, the reviewers and, most important, our authors.

These meticulous efforts coupled with the growth in numbers, eventually led to *BJR*|*Case Reports* obtaining the first impact factor (0.6) in the 2022 Journal Citation Reports which was awarded in June 2023, a most important recognition and a clear indicator of the quality and relevance of our journal.

Since its launch, the BIR and *BJR*|*Case Reports* have celebrated novel case reports published every year: the author of the best original case report, selected by the Publications Committee and *BJR*|*Case Reports* Editorial Board is nominated for the BIR Dr Prafulla Kumar Ganguli Award.

With this strong tradition and solid foundations, it is only natural that we look forward to the future and make plans for the continuous growth of our journal.

This new year, after gifting us with the new colourful journal cover, will also see the launch of a new initiative. Starting from 2024, *BJR*|*Case Reports* will publish Case Collections, the first two of which will be on “Imaging infectious diseases” and “Advances in nuclear medicine imaging”. These collections will grow over time, with the goal of becoming a go-to resource for clinical cases on a specific topic. We are sure that this initiative will be appreciated by both authors, who will gain more visibility, and readers, who will find cases of interest more easily.

Looking further into the future, I hope that our journal will always be considered as a reliable, reputable, high-quality destination for those who would like to publish a Case Report, Case Series or Technical Note, to share with colleagues their unusual cases or experience in diagnostic imaging and radiation oncology. From this exchange, we can all learn and benefit, and ultimately improve our practice.

